# Elevated resting heart rate is associated with the metabolic syndrome

**DOI:** 10.1186/1475-2840-8-55

**Published:** 2009-10-14

**Authors:** Ori Rogowski, Arie Steinvil, Shlomo Berliner, Michael Cohen, Nili Saar, Orit Kliuk Ben-Bassat, Itzhak Shapira

**Affiliations:** 1 The Departments of Medicine "D" & "E" and The Institute for Special Medical Examinations (MALRAM), Tel Aviv Sourasky Medical Center, Tel Aviv, Israel; 2 Sackler Faculty of Medicine, Tel Aviv University, Tel Aviv, Israel

## Abstract

**Background:**

Increased resting heart rate (RHR) may be associated with increased cardiovascular morbidity. Our aim was to explore the possibility that increased RHR is associated with the prevalence of the metabolic syndrome (MetS) in a sample of apparently healthy individuals and those with cardiovascular risk factors.

**Methods:**

We performed a cross-sectional analysis in a large sample of apparently healthy individuals who attended a general health screening program and agreed to participate in our survey. We analyzed a sample of 7706 individuals (5106 men and 2600 women) with 13.2% of men and 8.9% of the women fulfilling the criteria for the MetS. The participants were divided into quintiles of resting heart rate. Multiple adjusted odds ratio was calculated for having the MetS in each quintile compared to the first.

**Results:**

The multi-adjusted odds for the presence of the MetS increased gradually from an arbitrarily defined figure of 1.0 in the lowest RHR quintile (<60 beats per minute (BPM) in men and <64 BPM in women) to 4.1 and 4.2 in men and women respectively in the highest one (≥80 BPM in men and ≥82 BPM in women).

**Conclusion:**

Raised resting heart rate is significantly associated with the presence of MetS in a group of apparently healthy individuals and those with an atherothrombotic risk. The strength of this association supports the potential presence of one or more shared pathophysiological mechanisms for both RHR and the MetS.

## Background

There are multiple lines of emerging evidence which suggest that resting heart rate (RHR) is associated with the presence and/or the potential to develop cardiovascular disease [1-4]. We have currently analyzed the association between RHR and the presence of the metabolic syndrome (MetS), a recognized risk factor for cardiovascular disease [5,6]. A significant association might explain, at least in part, the observed link between RHR and cardiovascular disease. It can also raise the possibility of the existence of currently unknown shared pathophysiological mechanisms.

## Methods

### Study population

We have currently analyzed data which has been collected during the last five years from the Tel Aviv Medical Center Inflammation Survey (TAMCIS), a registered data bank of the Israeli Ministry of Justice [7-13]. This is a relatively large cohort of individuals who attended our medical centre for a routine annual check-up and gave their written informed consent for participation according to the instructions of the local ethics committee. A total of 14,888 subjects agreed to participate (9,412 males, 5,476 females). Multiple exclusions were conducted in order to analyze subjects without conditions that might influence heart rate or MetS parameters. Firstly, 2,657 subjects were excluded for having a current or past medical history of malignancy or immunosuppressive therapy, known inflammatory disease, pregnancy, steroidal or non-steroidal anti-inflammatory treatment, acute infection or having undergone any invasive procedures (surgery, catheterization, etc) during the prior 6 months. We further excluded 463 individuals with at least one proven past atherothrombotic event (myocardial infarction, cerebrovascular event or peripheral arterial disease) and 348 individuals due to absent recorded resting heart rate or any of the measurements of parameters relevant to the metabolic syndrome. In order to minimise the possible influence of medications on heart rate, we further excluded 930 individuals who were taking nitrates, alpha blockers, beta blockers, calcium channel blockers, antiarrhythmic drugs, thyroid replacement medications or digoxin. We excluded a further 1,131 individuals with anemia, defined as having an hemoglobin concentration below the lower normal limit according to our laboratory (135 g/l and 117 g/l for men and women respectively) and 1,653 currently smoking individuals.

### Determination of RHR

Baseline RHR was obtained manually at enrolment with one radial pulse measurement over a period of 60 seconds with the patient in a sitting position, after sitting in a quiet room for 5 minutes.

### Definition of MetS

The diagnosis of the metabolic syndrome was based on the National Cholesterol Education Program (NCEP) ATP III Criteria [14] with the modified Impaired Fasting Glucose criteria of the American Diabetes Association (ADA) [15] as proposed by the updated American Heart Association (AHA)/National Heart, Lung, and Blood Institute (NHLBI) scientific statement [16]. Accordingly, the criteria for MetS were based upon the existence of three or more of the following: (1) waist circumference > 88 cm in women or >102 cm in men; (2) a fasting triglyceride concentration >1.7 mmol/l; (3) an high density lipoprotein cholesterol (HDL-C) concentration <1.3 mmol/l in women or <1.0 mmol/l in men (4) a blood pressure above 130 mm Hg (systolic) or 85 mm Hg (diastolic) or use of antihypertensive drugs; and (5) a fasting plasma glucose >5.55 mmol/l or use of antidiabetic drugs.

### Laboratory methods

Blood was drawn during the morning hours following a fasting period of at least 12 hours using a standard Vacutainer gel tube (Becton Dickinson and company, New Jersey, USA). Triglycerides, HDL-C and glucose concentration were measured using a Bayer Advia 1650 chemistry analyzer and respective Bayer kits (Bayer healthcare diagnostics division, Newbury, UK). Blood pressure was obtained on two separate measurements following a five minute resting period.

### Statistical analysis

All data was summarized and displayed as mean (Standard deviation (SD)) for the continuous variables and as the number of patients (percentage) in each group for categorical variables.

In order to characterize the population we divided the patients of each gender into quintiles of resting heart rate and analyzed all results accordingly. For all categorical variables the Chi-Square test was used for assessing the overall statistical significance between the quintiles, while the One-Way Analysis of Variance was used for all continuous variables as well as for calculating the P value for the linear trend between the quintiles.

In order to better evaluate the magnitude of the association between each component of the MetS and the RHR, we calculated the estimated marginal means for the groups with and without each component, adjusting for age, former or never smoking status, alcohol consumption, exercise activity, oral temperature, family history of premature cardiovascular disease (CVD), use of aspirin as well as the presence of all four others components of the MetS, using analysis of covariance (ANCOVA) under a general linear model.

Finally, in order to quantify the relative odds of having the MetS as a function of the quintiles of RHR, we arbitrarily defined the lowest quintile as 1.0 and calculated the adjusted odds ratio (OR) for having the MetS for each of the higher quintiles with adjustment for age, former or never smoking status, alcohol consumption, exercise activity, oral temperature, family history of CVD and the use of aspirin, using logistic regression. All above analyses were considered significant at p < 0.05 (two tailed). The SPSS statistical package was used to perform all statistical evaluation (SSPS Inc., Chicago, IL, USA).

## Results

**Figure 1:**
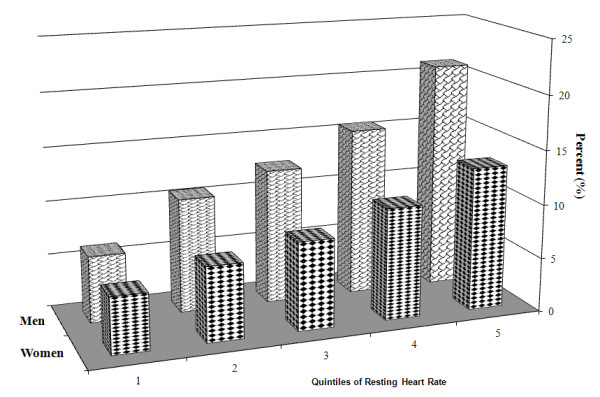
**Percentage of individuals fulfilling the criteria for the metabolic syndrome in each quintile of resting heart rate**.

**Figure 2:**
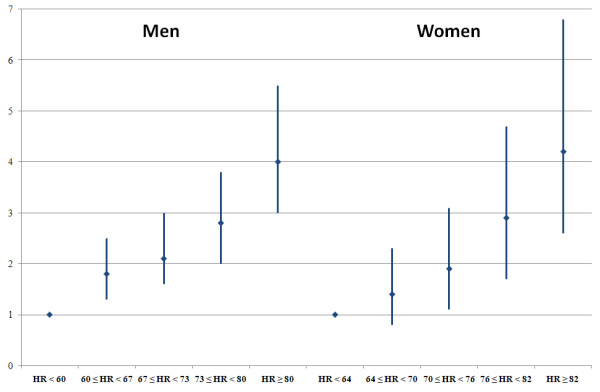
Multi-Adjusted Odds Ratio (95% Confidence Interval) for having the Metabolic Syndrome.

We have currently performed a cross-sectional analysis regarding the presence or absence of the MetS in a sample of 5,106 men and 2,600 women with respective mean (SD) ages of 43 (11) and 44 (11) years. This cohort included a total of 674 males and 231 females defined as having the MetS. The baseline characteristics of both men and women in relation to quintiles of RHR are reported in Tables 1 and 2, respectively. It can be seen that the prevalence of the MetS increases with the elevation of RHR in both men and women. In fact, the prevalence of MetS was found to be 6.2% and 5.2% (for men and women, respectively) in the first quintile of RHR and this increased up gradually to the respective percentages of 21.1% and 13.3% in the fifth quintile (Figure 1). With the exception of HDL cholesterol, a significant association was noted between RHR and each of the components of the MetS for both genders (Table 3). Finally, we have calculated the crude and multi-adjusted odds for the presence of MetS in both genders according to quintiles of the RHR (Table 4 and Figure 2). Again, a clear and sequential increment is seen for the odds of having MetS according to the quintiles of the RHR in both genders.

**Table 1:** Characteristics* of men according to quintiles of resting heart rate

**Men (N = 5,106)**	**1^st ^Quintile**	**2^nd ^Quintile**	**3^rd ^Quintile**	**4^th ^Quintile**	**5^th ^Quintile**	**ANOVA**	**P for linear trend**
	**HR < 60**	**60 ≤ HR < 67**	**67 ≤ HR < 73**	**73 ≤ HR < 80**	**HR ≥ 80**	**P Value**	
	**n = 947**	**n = 1137**	**n = 1020**	**n = 1009**	**n = 993**		
Age (years)	43 (11)	44 (11)	44 (11)	43 (11)	42 (10)	0.080	0.135
Waist (cm)	92 (9)	94 (9)	95 (11)	96 (10)	96 (11)	< 0.001	< 0.001
BMI (kg/m^2^)	25.8 (3.0)	26.4 (3.2)	26.8 (3.7)	27.0 (3.8)	27.3 (4.1)	< 0.001	< 0.001
Diastolic BP (mmHg)	76 (7)	77 (7)	78 (8)	78 (8)	80 (9)	< 0.001	< 0.001
Systolic BP (mmHg)	121 (12)	123 (14)	123 (14)	123 (14)	127 (15)	< 0.001	< 0.001
Alcohol consumption (glasses/week)	1.4 (2.2)	1.4 (2.5)	1.1 (1.7)	1.2 (2.4)	0.8 (1.6)	< 0.001	< 0.001
Physical exercise (hours/week)	3.3 (3.1)	2.4 (2.6)	2.3 (2.7)	2.0 (2.5)	1.8 (2.4)	< 0.001	< 0.001
Smoking status, n(%)							
Former	305 (32.2)	347 (30.5)	309 (30.3)	271 (26.9)	268 (27.0)	0.031	
Never	642 (67.8)	790 (69.5)	711 (69.7)	738 (73.1)	725 (73.0)		
Hypertension, n (%)	123 (13.0)	196 (17.2)	200 (19.6)	203 (20.1)	287 (28.9)	< 0.001	
Metabolic Syndrome, n (%)	59 (6.2)	121 (10.6)	127 (12.5)	157 (15.6)	210 (21.1)	< 0.001	
Glucose (mmol/l)	5.08 (0.64)	5.15 (0.69)	5.19 (0.79)	5.26 (0.96)	5.46 (1.31)	< 0.001	< 0.001
HDL Cholesterol (mmol/l)	1.36 (0.29)	1.31 (0.26)	1.31 (0.26)	1.29 (0.26)	1.28 (0.23)	< 0.001	< 0.001
LDL Cholesterol (mmol/l)	3.09 (0.78)	3.18 (0.80)	3.19 (0.82)	3.19 (0.84)	3.22 (0.83)	0.009	0.001
Triglycerides (mmol/l)	1.08	1.21	1.25	1.34	1.38	< 0.001	< 0.001

* data presented as arithmetic mean (standard deviation) for continuous variables (with the exception of triglycerides which are presented as geometric mean) and number of individuals (percentage) for dichotomous variables.

** list of abbreviations: HR = heart rate; ANOVA = analysis of variance; BMI = body mass index; BP = blood pressure; HDL = high-density lipoprotein; LDL = low density lipoprotein.

**Table 2:** Characteristics* of women according to quintiles of resting heart rate

**Women (n = 2,600)**	**1^st ^Quintile**	**2^nd ^Quintile**	**3^rd ^Quintile**	**4^th ^Quintile**	**5^th ^Quintile**	**ANOVA**	**P for linear trend**
	**HR < 64**	**64 ≤ HR < 70**	**70 ≤ HR < 76**	**76 ≤ HR < 82**	**HR ≥ 82**	**P Value**	
	**n = 518**	**n = 519**	**n = 571**	**n = 454**	**n = 538**		
Age (years)	47 (10)	46 (10)	44 (10)	42 (10)	41 (11)	< 0.001	< 0.001
Waist (cm)	80 (11)	81 (11)	81 (11)	83 (13)	82 (13)	0.006	< 0.001
BMI (kg/m^2^)	24.5 (3.9)	24.8 (4.1)	25.0 (4.2)	25.6 (5.3)	25.3 (5.1)	0.002	< 0.001
Diastolic BP (mmHg)	73 (7)	73 (8)	73 (8)	74 (8)	75 (9)	< 0.001	< 0.001
Systolic BP (mmHg)	115 (14)	116 (15)	115 (15)	116 (15)	118 (16)	0.003	0.001
Alcohol consumption (glass/week)	0.7 (1.6)	0.5 (1.3)	0.6 (1.3)	0.5 (1.2)	0.4 (1.0)	0.007	0.006
Physical exercise (hours/week)	2.7 (2.8)	2.0 (2.4)	1.7 (2.4)	1.7 (2.5)	1.6 (2.6)	< 0.001	< 0.001
Smoking status, n(%)							
Former	158 (30.5)	128 (24.7)	140 (24.5)	101 (22.2)	105 (19.5)	0.001	
Never	360 (69.5)	391 (75.3)	431 (75.5)	353 (77.8)	433 (80.5)		
Hypertension, n (%)	45 (8.7)	56 (10.8)	50 (8.8)	55 (12.1)	81 (15.1)	0.004	
Metabolic Syndrome, n (%)	27 (5.2)	36 (6.9)	47 (8.2)	47 (10.4)	74 (13.3)	< 0.001	
Glucose (mmol/l)	4.89 (0.52)	4.90 (0.51)	5.00 (0.68)	5.01 (0.87)	5.14 (0.91)	< 0.001	< 0.001
HDL Cholesterol (mmol/l)	1.72 (0.39)	1.70 (0.37)	1.68 (0.37)	1.66 (0.37)	1.68 (0.40)	0.165	0.031
LDL Cholesterol (mmol/l)	3.07 (0.83)	3.09 (0.86)	3.06 (0.87)	3.03 (0.82)	3.02 (0.82)	0.635	0.165
Triglycerides (mmol/l)	0.89	0.96	1.03	1.04	1.11	< 0.001	< 0.001

* data presented as arithmetic mean (standard deviation) for continuous variables (with the exception of triglycerides which are presented as geometric mean) and number of individuals (percentage) for dichotomous variables.

** list of abbreviations: HR = heart rate; ANOVA = analysis of variance; BMI = body mass index; BP = blood pressure; HDL = high-density lipoprotein; LDL = low density lipoprotein.

**Table 3:** Estimated marginal mean* (95% confidence interval) of resting heart rate in relation to each component of the MetS

**Men**	**Component absent**	**Component present**	**P Value**
Hypertension	71.1 (70.0-72.2)	74.5 (73.4-75.6)	< 0.001
Waist circumference	71.5 (70.5-72.5)	74.1 (72.9-75.3)	< 0.001
IFG	71.0 (69.9-72.0)	74.7 (73.5-75.8)	< 0.001
Triglycerides	71.9 (70.8-72.9)	73.8 (72.6-74.9)	< 0.001
HDL Cholesterol	72.8 (71.8-73.8)	72.8 (71.5-74.1)	0.966

			
Women			

Hypertension	74.6 (72.6-76.5)	77.8 (75.8-79.8)	< 0.001
Waist circumference	75.5 (73.5-77.4)	76.9 (75.0-78.9)	0.011
IFG	74.9 (73.0-76.8)	77.5 (75.4-79.6)	< 0.001
Triglycerides	75.0 (73.1-76.9)	77.4 (75.3-79.5)	< 0.001
HDL Cholesterol	76.0 (74.1-77.9)	76.4 (74.3-78.5)	0.524

*All means are adjusted for age, former or never smoking status, family history of CVD, aspirin usage, alcohol consumption, measured oral temperature and physical exercise, and in addition, to the presence of all other components of the MetS.

** list of abbreviations: MetS = metabolic syndrome; IFG = impaired fasting glucose; HDL = high-density lipoprotein.

**Table 4:** OR (95% CI) for having the MetS according to quintiles of resting heart rate

	**1^st ^Quintile**	**2^nd ^Quintile**	**3^rd ^Quintile**	**4^th ^Quintile**	**5^th ^Quintile**
Men	HR < 60	60 ≤ HR < 67	67 ≤ HR < 73	73 ≤ HR < 80	HR ≥ 80
	n = 947	n = 1137	n = 1020	n = 1009	n = 993

Crude OR	1.00	1.8 (1.3-2.5)^§^	2.1 (1.6-3.0)^§^	2.8 (2.0-3.8)^§^	4.0 (3.0-5.5)^§^
Multiadjusted* OR	1.00	1.9 (1.3-2.7)^§^	2.1 (1.4-2.9)^§^	2.8 (2.0-4.0)^§^	4.2 (3.0-5.9)^§^

					
Women	HR < 64	64 ≤ HR < 70	70 ≤ HR < 76	76 ≤ HR < 82	HR ≥ 82
	n = 518	n = 519	n = 571	n = 454	n = 538

Crude OR	1.00	1.4 (0.8-2.3)	1.6 (1.0-2.7)	2.1 (1.3-3.4)^§^	2.9 (1.8-4.6)^§^
Multiadjusted* OR	1.00	1.2 (0.7-2.2)	1.9 (1.1-3.3)^†^	2.9 (1.7-4.9)^§^	3.6 (2.2-6.1)^§^

† - 0.05 > P ≥ 0.01; § - P < 0.01

* Adjusted for age, former or never smoking status, family history of CVD, aspirin usage, alcohol consumption, measured oral temperature and physical exercise.

** list of abbreviations: MetS = metabolic syndrome; OR = odds ratio; CI = confidence interval; HR = heart rate.

## Discussion

We have presently documented a relatively strong association between RHR and the presence of the MetS. Such a strong association might suggest that there is a shared pathophysiological pathway which is yet to be revealed. We believe therefore, that RHR is an extremely easy-to-perform and almost costless parameter which may improve the early detection of cardiovascular risk. Of special note might be the fact that even a small increment in RHR (for example from the first to the second quintile) had a clear influence on the odds of having MetS. Moreover, the prevalence of the MetS was three times higher in individuals in the fourth quintile and this was still within the "normal" RHR that is - to date - accepted as being between 60 and 80. Again, this might be another observation to support the notion that one should look at RHR as a continuous variable, putting into perspective the currently used normal limits.

Sympathetic overactivity or parasympathetic underactivity might underly the aforementioned observation. In fact, sympathetic imbalance has been described for several of the components of the MetS including hypertension [17], waist circumference [18-21], impaired fasting glucose [22,23] as well as hypertriglyceridemia [24]. Deniz et al [25], demonstrated a significant difference in RHR between MetS and controls (105 vs. 88, P < 0.001). That however, was a small case-control study involving 64 young male subjects with MetS and 40 overweight matched control subjects without MetS. In addition, Deniz et al found a significant association between the presence of the MetS and impaired heart rate recovery and low exercise capacity [25]. Thus, one could argue that our observation is an expected one although the strength of the association was not known. Our study is significant, therefore, in the determination of the strength of the association, a strength that could support shared pathophysiological mechanisms. These suggested mechanisms might work in both directions since the treatment of the dysmetabolism might have a favorable effect on the heart rate [26].

In a previous study which solely involved men, we addressed the question of the association between RHR and the presence of microinflammatory changes [10]. It is known that individuals with the MetS do harbor a low grade inflammation but the presence of MetS in relation to RHR was not explored in that study.

Resting heart rate measurements are known to be influenced by multiple factors. In the current study design, although there were multiple exclusions and statistical adjustments performed, we could not account for every factor. Thus we acknowledge several limitations to the present study. Firstly, the RHR was obtained by single measurements following five minutes of rest and not by repeated measurements. Since all of the analyses were based on this single measurement and since unconditioned individuals might require more time to return to their usual heart rate at rest, it is possible that our results were accordingly influenced. On the other hand, we believe that the large number of individuals analysed dilutes this possible effect and makes it unlikely that repeated measurements or longer resting prior to measurement would influence our results significantly. Secondly, we did not exclude patients with abnormal thyroid function, mainly because the survey does not include those measurements. We did however, exclude all individuals reporting thyroid disease or taking thyroid replacement therapy. Thirdly, we did not measure insulin levels and thus could not calculate the HOMA index. Lacking this objective laboratory parameter, we could only assume that there is a shared pathophysiological pathway and that future studies in this field might reveal what it is. Fourthly, the individuals were not evaluated routinely by echocardiography and thus we were not able to exclude individuals with a cardiomyopathy, including such a cardiomyopathy secondary to tachycardia. Due to the relatively healthy population evaluated and the large number of individuals we believe that the number of individuals with a significant cardiomyopathy included is negligible and would therefore not have influenced the results significantly. Finally, we did not exclude patients regularly performing sport activity, although we did adjust our results to their levels of physical activity. In addition, since our population is mainly referred from places of employment, there exists the possibility of "healthy worker" selection bias and thus generalization of the results might be limited.

The question of "normalcy" for RHR remains. Our study does support, at least in relation to the presence of the MetS, the finding that the lower the RHR is, the lower the dysmetabolic risk. This information might be relevant if new "normal values" for RHR are to be determined in the future.

## Competing interests

The authors declare that they have no competing interests.

## Authors' contributions

OR and AS have participated in the design of the study, performed the statistical analyses and drafted the paper. SB and SI conceived the study, participated in its design and coordination and helped to draft and review the manuscript. MC, NS and OKB helped in the data organization and retrieval, English editing and final draft preparation. All of the authors have read and approved the final manuscript.
